# Cloning and characterization of miRNAs from maize seedling roots under low phosphorus stress

**DOI:** 10.1007/s11033-012-1661-5

**Published:** 2012-05-05

**Authors:** Zhiming Zhang, Haijian Lin, Yaou Shen, Jian Gao, Kui Xiang, Li Liu, Haiping Ding, Guangsheng Yuan, Hai Lan, Shufeng Zhou, Maojun Zhao, Shibin Gao, Tingzhao Rong, Guangtang Pan

**Affiliations:** 1 Key Laboratory of Biology and Genetic Improvement of Maize in Southwest China, Ministry of Agriculture, Maize Research Institute, Sichuan Agricultural University, Chengdu Campus, 211 Huimin Road, Wenjiang, 611130 Sichuan China; 2 Sichuan Agricultural University, Chengdu Campus, 211 Huimin Road, Wenjiang, 611130 Sichuan China; 3 College of Life and Basic Sciences, Sichuan Agricultural University, 46 Xinkang Road, Ya’an, 625014 Sichuan China

**Keywords:** miRNAs, Target gene, Expression pattern, Phosphorus stress, Maize

## Abstract

MicroRNAs (miRNAs) are a class of small, non-coding regulatory RNAs that regulate gene expression by guiding target mRNA cleavage or translational inhibition in plants and animals. In this study, a small RNA library was constructed to identify conserved miRNAs as well as novel miRNAs in maize seedling roots under low level phosphorus stress. Twelve miRNAs were identified by high throughput sequencing of the library and subsequent analysis, two belong to conserved miRNA families (miRNA399b and miRNA156), and the remaining ten are novel and one of latter is conserved in gramineous species. Based on sequence homology, we predicted 125 potential target genes of these miRNAs and then expression patterns of 7 miRNAs were validated by semi-RT-PCR analysis. MiRNA399b, Zma-miR3, and their target genes (*Zmpt1* and *Zmpt2*) were analyzed by real-time PCR. It is shown that both miRNA399b and Zma-miR3 are induced by low phosphorus stress and regulated by their target genes (*Zmpt1* and *Zmpt2*). Moreover, Zma-miR3, regulated by two maize inorganic phosphate transporters as a newly identified miRNAs, would likely be directly involved in phosphate homeostasis, so was miRNA399b in *Arabidopsis* and rice. These results indicate that both conserved and maize-specific miRNAs play important roles in stress responses and other physiological processes correlated with phosphate starvation, regulated by their target genes. Identification of these differentially expressed miRNAs will facilitate us to uncover the molecular mechanisms underlying the progression of maize seedling roots development under low level phosphorus stress.

**Electronic supplementary material:**

The online version of this article (doi:10.1007/s11033-012-1661-5) contains supplementary material, which is available to authorized users.

## Introduction

Phosphorus is one of the most essential and limiting macronutrients for plants correlated with energy metabolism, biosynthesis of nucleic acids, and membranes. It is also available for plant uptake because of adsorption, precipitation, or conversion to organic forms [1]. Phosphate (Pi) deficiency could affect crop productivity seriously and diminish yields in agriculture around the world, especially in acidic soils of tropical and subtropical regions. However, during the course of evolution, plants have possessed a wide range of morphological and molecular mechanism adapting to increase remobilization, uptake, and efficient use of Pi in Pi deficiency condition [1–3]. How to cope with this problem? Unveiling the molecular mechanism under the Pi starvation responses of plants will be helpful to solve this issue [4, 5]. Recently, it is shown that miR399 was highly induced, whereas the target UBC mRNA was reduced by low-Pi stress in Arabidopsis under Pi starvation. MiRNA399 targets at two genes belonging to two different gene families: Pi transporter [6] and ubiquitin conjugating enzyme which (UBC24) was involved in protein degradation pathway. UBC24 can cause the accumulation Pi supply activated by the Pi starvation [7]. Sunkar [8] has revealed a mechanism by which plants regulate inorganic Pi homeostasis to adapt to environmental changes in Pi availability. However, only few miRNAs and their target genes were reported to be directly involved in response to low phosphorus stress.

MicroRNAs (miRNAs) are a class of non-coding RNA gene whose products are ~22 nt sequences that play important roles in the regulation of translation and degradation of mRNAs. Since the discovery of the founding members of the class, *let*-*7* and *lin*-*4* miRNAs in *Caenorhabditis elegans* [9], many miRNAs have been found in animals and plants [10]. In plants, miRNAs are processed from stem-loop regions of RNA transcripts by a dicer-like enzyme, and RNA generates a pre-miRNA, which is processed by DCL1 and possibly other proteins to yield the fold-back precursor pre-miRNA. The pre-miRNA may be trafficked out of the nucleus by HST (Exportin-5) or it may be retained in the nucleus, where HEN1, HYL1, and DCL1 are localized. These proteins process the pre-miRNA further to form a miRNA–miRNA imperfect duplex, which is unwound and loaded on to the RISC (HYL1 and DCL1 may act together here), and lastly the RISC complex is guided by the miRNA to the target mRNA, possibly by a helicase scanning mechanism [11]. In plants, most miRNAs regulate target gene expression via mRNA degradation [11]. MiRNAs recognize completely or partially complementary sequences in target mRNAs and guide them to cleavage or translational arrest, and plant miRNAs usually recognize one motif in the coding region of their targets and affect their stability. It is thought that the better complementary between plant miRNAs and their targets, the more favorable the latter mechanism and plant miRNAs can regulate a variety of biological processes, such as root growth flower and leaf development. Moreover, recently discovered miRNAs have emerged as important players in plant stress responses, such as drought stress, mechanical stress, pathogen infection [12], heavy metal stress, and nutrition deficiency. The fact that some of the miRNAs are up- or down-regulated in response to stress implies that these miRNAs have a role in stress tolerance. Stress-induced miRNAs might down-regulate their target genes, which may encode negative regulators of stress responses. Conversely, miRNAs caused the accumulation of their target mRNAs and down-regulated in response to stress, which may contribute positively to the adaptation to stress.

Maize is one of the most important crops, which one-third of the people in the world feed on. Little is known about the regulatory systems supervising such complex processes as how Pi starvation-responsive genes are regulated and how plants coordinate Pi acquisition and allocation to maintain Pi homeostasis during Pi starvation in maize. There are six loci in maize for miRNA399 family, which was predicted by computational methods. However, there is no report of miRNA399 and phosphorus homeostasis in maize so far. In this study, miRNA399b was obtained by cloning method, and its expression and target gene analysis showed that it may be followed by a similar homeostatic regulatory mechanism in Arabidopsis and Maize mediated by miRNA399 under low phosphorus stress.

## Materials and methods

### Plant material and growth conditions

Maize elite inbred line 178, with high absorption efficiency of phosphorus, provided by Maize Research Institute, Sichuan Agricultural University, was used in this study. The seeds were surface-sterilized and germinated in quartz granule in a growth chamber at 26 °C with a 16 h light/8 h dark photoperiod cycle for 8 days with half-strength modified Hoagland nutrient solution containing 1 mM KH_2_PO_4_ (high Pi) or 10 μM KH_2_PO_4_ (low Pi), and then transferred into individual plastic pots for 3 days without endosperm. For short-term sulfate starvation treatment, 2-day-old plants were transplanted in high-Pi medium for the indicated times and then transferred to Pi-deficient nutrient solution which was subsequently grown for 12, 24, 48, 72, and 96 h. As a control experiment, plants were transferred to +Pi nutrient solution and cultured for the same length of time. And then the roots were collected at 0 (CK), 12, 24, 48, 72, and 96 h after the onset of stress.

### Isolation and cloning of miRNAs

Total RNA was isolated from the roots using TRizol (Invitrogen), and then treated with RNAase-free DNAase I according to the manufacturer’s instructions. A small pooled RNA library was constructed using mirVana^™^ miRNA Isolation Kit (Ambion). Cloning of miRNAs was performed using miRNA cloning kit (Takara). Small RNAs (<200 nt) were separated on a denaturing 15 % polyacrylamide gel. Molecules ranging in size from 18 to 26 nt were excised and recovered using 30 μl RNAase-free water. The recovered small RNAs were ligated sequentially with a 3′ and 5′ adapters. Chimeric RNA/DNA adapters were purified by 10 % denaturing polyacrylamide gel electrophoresis; the small RNAs ranging in size from 62 to 70 nt were eluted from the gel. Reverse transcription was performed using the adapter primers, and the recovered DNA amplification product was directly transformed into pMD18-T vector (Takara) for plasmid cultivation. Plasmids were isolated from individual colonies for amplified cultivation, sequenced and processed for BLAST analysis against the NCBI genomic data sets for maize and other gramineous species.

### Sequence analysis and prediction of fold-back structures

All sequences were used to search the Rfam (www.sanger.ac.uk/Software/Rfam) database with BLASTN [13],to identify sequence tags originated from coding exons, repeats, rRNA, tRNA, snRNA, and snoRNA, which were removed from the small RNA sequences, and the remaining sequences were compared against rice and Arabidopsis ncRNAs deposited in the NCBI GenBank database and Rfam 8.0 database. To determine whether these small RNA sequences from *Zea mays* L. are considered as candidate miRNAs, these cleaned small RNA sequences described above were mapped to the draft *Zea mays* L. (ZmB73 RefGen_v2, November 2010) genome sequences using SOAP (short oligonucleotide alignment program) software (http://soap.genomics.org.cn). Only the miRNAs that perfectly mapped onto the genome were considered in the current study. To identify potential miRNA genes, the MIREAP algorithm (http://sourceforge.net/projects/mireap) was employed to obtain all candidate precursors with hairpin-like structures that were perfectly mapped by sequencing tags. Candidate miRNAs’ sequences with perfect matches against these sets were used for fold-back secondary structure prediction, which was conducted on web-based program Mfold 3.1 [14], according to the criteria described by Ambros [15].

### Target gene prediction

In order to indentify the accuracy of the target genes, we adopted a set of rules proposed in earlier reports for predicting miRNA targets [16, 17]. These criteria are as follows: allowing one mismatch in the region complementary to nucleotide positions 2–12 of the miRNA, but not at position 10/11, and three additional mismatches between positions 12 and 22 but with no more than two continuous mismatches. Therefore, candidate miRNA target genes were determined using publicly available prediction algorithms, including psRNA (http://plantgrn.noble.org/psRNATarget/ and WMD3 (http://wmd3.weigelworld.org/cgi-bin/webapp.cgi?) target program with default parameters. Newly identified maize miRNA sequences were used as custom miRNA sequences, *Zea mays* L. (maize) DFCI Gene Index (ZMGI) Release 19 transcript/genomic library and *Zea mays* L. ZmB73 v4a.53 (MGC)/*Zea mays* EST ZmGI-18.0 (GeneIndex) were used as custom plant databases.

### Expression analysis of miRNAs by poly (A)-tailed RT-PCR

A 5 μg sample of total RNA was used for cDNA synthesis using the Invitrogen Reverse Transcription reagents kit (Invitrogen, USA). Gene-specific primers were designed using Primer Express version 2.0 (Applied Biosystems, USA) and synthesized in Sangon Biotech Co. (Sangon, Shanghai China). Primers used in the real-time PCR and one common adaptor primer (5′-CGAACATGTACAGTCCATGGATAG-3′) are listed in Table 1. Relative quantitative analysis was performed using an Applied Biosystems 7900HT (Applied Biosystems, USA) under the following conditions: 94 °C/3 min (one cycle), 94 °C/30 s, 60 °C/30 s, 72 °C/30 s (40 cycles). Transcript abundances were identified using SYBR Green PCR Master Mix (Applied Biosystems, USA). Each reaction contained 1× mix buffer, 0.25 μM each primer, and about 2 ng cDNA in a final volume of 20 μl. Three replicates were employed for each tested sample and template-free negative controls. Mitochondrial 5S RNA was used as an internal control to normalize all data. Melting curves were performed on the product to test if only a single product was amplified without primer-dimers and other bands. The resulting products with all primer combinations were initially visualized on 2 % agarose gel to confirm the generation of a single product of the correct size.

**Table 1:** Known and novel cloned miRNAs obtained from maize seedling roots under low phosphorus stress

Known or putative miRNAs	Sequence (From 5′–3′)	Length	Fold-back	Length of primary miRNA
Zm-miRNA1	UUGUUUGGAAUUAUAAUCUGC	21	Yes	131
Zm-miRNA2	AGAGGGGGAUUGGAGGGGAUU	21	Yes	101
Zm-miRNA3	UUGGUGACCAGGGAAAUGGAG	21	Yes	123
Zm-miRNA4	UGAGAGAAUGGUAUAAUCACA	21	Yes	188
Zm-miRNA5	UGAAGGGGAUUGGAGAGGAU	20	Yes	184
Zm-miRNA6	UCUAAAAUGAGUGGUGCUGAU	21	Yes	172
Zm-miRNA7	AAUAUUAGACAGAAAAGUUAG	21	Yes	70
Zm-miRNA8	GCUCGGCAAAGACUGACGGCC	21	Yes	77
Zm-miRNA9	UAGCCAGGGAUGAUUUGCCUG	21	Yes	122
Zm-miRNA10	UUCUGAUGUUCAUGAGCUGGCU	22	Yes	121
miRNA399b	UGCCAAAGGAGAGCUGUCCUG	21	Yes	
miRNA156	UGACAGAAGAGAGUGAGCAC	20	Yes	

### Expression patterns of miRNA399b and Zma-miR3

To indentify the expression patterns of candidate miRNAs, real-time PCR analysis was performed. The corresponding sequences for miRNA399b (5′-UGCCAAAGGAGAGCUGUCCUG-3′) and Zma-miR3 (5′-UUGGUGACCAGGGAAAUGGAG-3′) were used as the forward primers, and the 3′-common primer (Qiagen), as the reverse primer. The analytical procedure of Real-time PCR analysis was mentioned above (see the method in Expression analysis of miRNAs by poly (A)-tailed RT-PCR).

### The structure and expression patterns of *Zmpt1* and *Zmpt2*

Using microarray analysis, we found that maize inorganic Pi transporters 1 and 2 are induced by low phosphorus stress (data not shown). We used DNAman software to analyze the homology and the conserved target binding region of the Pi transport proteins among *Arabidopsis*, rice, and maize (*Zmpt1* and *Zmpt2*). To monitor the expression patterns of *Zmpt1* and *Zmpt2*, maize seedling roots were collected for RNA isolation at 0 (CK), 12, 24, 48, 72, and 96 h, discarding Pi from the growing media. We used DNAse-treated RNA for cDNA synthesis by reverse transcription PCR with SYBR^®^ PrimeScript^™^ RT reagent kit (Takara). The PCR reactions were performed in a final volume of 20 μl with 0.5 μg of RNA.

The expression patterns of *Zmpt1* and *Zmpt2* were examined by real-time PCR. The probes were designed with Primer 6.0 program and verified by RT-PCR. Only the primers with identical size amplicons were selected for further real-time PCR analysis. The sequences 5′-ACGCCTTCACCTTCTTCTTCG-3′ and 5′-CAGGACAGGAGCAAGACGGA-3′ were used as forward and reverse primers, respectively, for amplification of *Zmpt1*. 5′-CGCATCATCCTCATCCTGG-3′ and 5′-CCAAGAACGCCAAGCAGGC-3′ were used for amplification of *ZmPT2* and 5′ -GTTGGGCGTCCTCGTCA-3′ and 5′-TGGGTCATCTTCTCCCTGTT-3′, for Actin. The analytical procedure of real-time PCR analysis was mentioned above (see the method in Expression analysis of miRNAs by poly (A)-tailed RT-PCR).

## Results

### The construction of small RNA libraries and sequence information

To indentify the known and novel miRNAs involved in response to low phosphorus stress, we constructed a small pooled RNA library (RNA sizes from 16 to 30 nt) from maize seedling roots. More than 5,000 clones were selected from this library for bacterial PCR detection and 245 clones ranging in size from 19 to 24 nt were obtained for further sequencing and fold-back structure prediction. For validation of candidate miRNAs, five cDNA libraries were constructed by poly (A)-tailed RT-PCR, using maize seedling roots subjected to the stress conditions. These libraries are suitable for further validation of newly isolated and known miRNAs and for expression pattern analysis by semi-quantitative RT-PCR and real-time PCR.

**Fig. 1:**
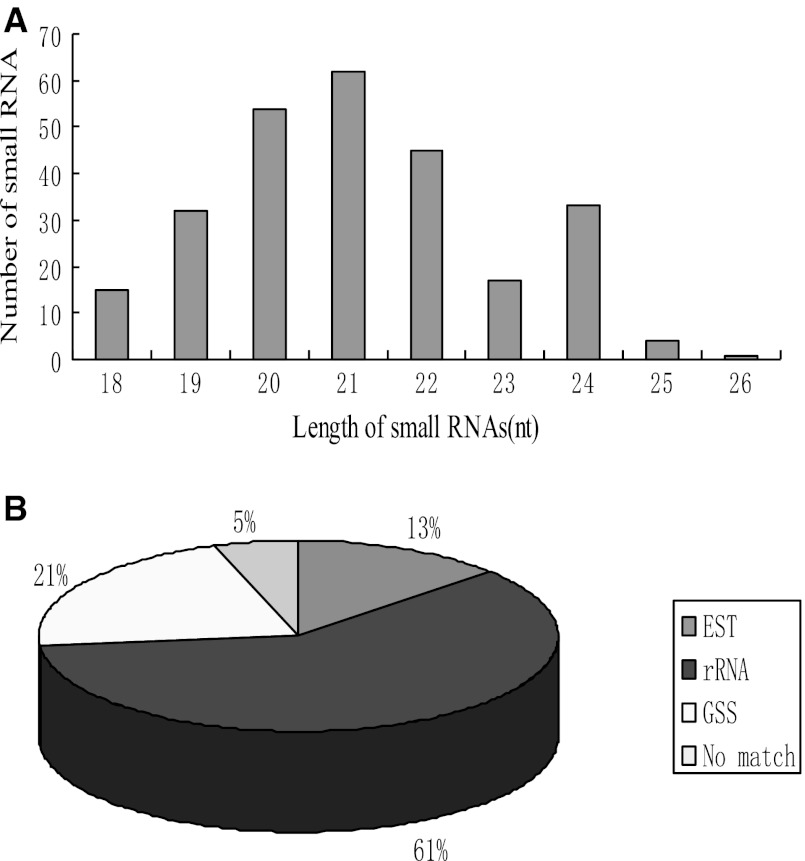
Frequency distribution of small RNA with different length and the pie chart of annotation for all sRNAs sequence using BLASTN searches. **a** Size distribution of small RNAs sequences cloned from Maize. **b** The proportion of sequences matched to maize genomic data sets

Two hundred and sixty-three sequences were obtained ranging in size from 18 to 24 nt. BLASTN searches revealed that 159 (61 %) of these sequences were known rRNAs. Ninety (21 %) sequences were well matched against maize genomic and EST data sets. However, the remaining 14 sequences were not matched, either in maize or related gramineous species genomic and EST data sets, these sequences were excluded from further analysis. The size distribution and BLASTN information are listed in Fig. 1a, b.

### Identification of two known and ten novel maize miRNAs

**Fig. 2:**
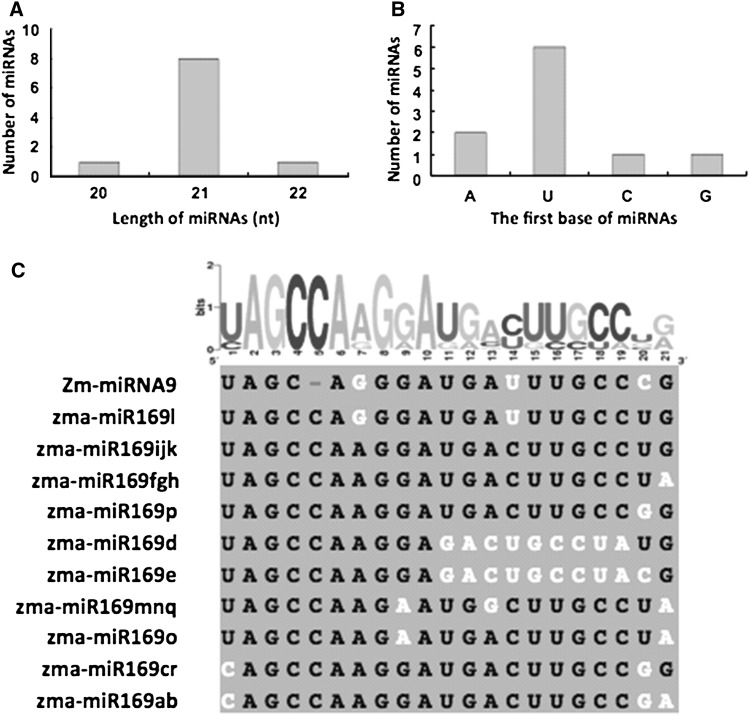
**a** The length distribution of newly identified miRNAs. **b** The number of miRNAs beginning with identified miRNAs. **c** Sequences of newly identified Zm-miR9 and known Zm-miRNA169 family members on the *left,* the different bases are shown in *white*. On the *right hand side* the frequency of different bases along Zm-miR9 and Zm-miRNA169 family members sequences

Ninety sequences were selected for miRNA prediction, of which 12 maize miRNAs were identified, including ten new miRNAs, found by sequence similarity searches against miRBase (http://www.mirbase.org/search.shtml) (Table 1). Two miRNAs sequences were identical to Zma-miRNA399 and Zma-miRNA156, which had been computationally predicted before. We also found a miRNA whose sequence was similar to the known maize miRNA169 family members (Table 1, Zma-miR09). The new miRNAs belong to one size class: 21 nt in length (Table 1; Fig. 2a). Six of the ten newly identified miRNAs begin with a 5′ uridine, which is a characteristic feature of miRNAs (Table 1; Fig. 2b).

So far, 18 members of miRNA169 family (a–r) were deposited in miRBase web site (http://www.mirbase.org/). All the Zma-miRNA169 family is identical with 21 base pairs. The Zma-miRNA169 family sequences are shown in Fig. 2c. Zma-miRNA169a, Zma-miRNA169b, Zma-miRNA169c, and Zma-miRNA169r begin with C while others have U as the first nucleotide. Zma-miRNA169a and Zma-miRNA169b have identical sequences while differing from Zma-miRNA169c and Zma-miRNA169r only at the last position (3′). Zma-miRNA169d and Zma-miRNA169e differ only at position 20, but there are larger differences than the rest members of the family. Zma-miRNA169f, Zma-miRNA169g, and Zma-miRNA169h sequences are identical and only differ from Zma-miRNA169ijk at the last position. Zm-miRNA9 just has 20 nt length, there has one “C” nucleotide deletion in the position 4 or 5, and just has one nucleotide different with Zma-miR169l (Fig. 2c).

### Precursors, fold-back structures and conservation analysis of the newly cloned miRNAs

We obtained ten precursors from maize genomic data set using the newly cloned sequences, with the length between 70 and 180 nt (Table 1). All the sequences were capable of forming stable stem-loop structures characteristic for miRNAs (Supplemental Fig. S1). To establish whether the ten newly cloned miRNAs are conserved in maize and other gramineous species, we used their sequences to search the genomic data of gramineous species (rice, wheat, barley, sorghum, and sugarcane). We found that one of them, Zma-miR2, is conserved among maize, rice, wheat, and sorghum. However, there are single base-pair differences. The conserved fold-back structures of Zma-miR2 in gramineous species are shown in (Supplemental Fig. S2).

### Target gene prediction

One hundred and twenty-five putative target genes were identified, of which, 80 were regulated by ten newly cloned miRNAs. The average number of predicted targets per miRNA was eight and varied greatly among them, from none for Zma-miR10 to 24 for Zma-miR2. Zma-miR4 had only one predicted target. Two known miRNAs of miRNA156 family were complementarily integrated with SBP family protein genes, which have been predicted and validated in rice and *Arabidopsis* [6] but not reported in maize. Another known maize miRNA (miR399b) negative regulated inorganic Pi transport protein genes, which were directly involved in maintaining Pi homeostasis in rice and *Arabidopsis*. Moreover, the putative novel miRNA Zma-miR3 also combined with the inorganic transport proteins’ genes. Are the functions of the two miRNA similar? The answer would be based on further research. In addition, several stress response genes were predicted as target genes of some new miRNAs (for example: SOD, Thioredoxin for Zma-miR2, 5 and GST for Zma-miR6), which might indicate these miRNAs directly involvement in Pi starvation stress response and some transcription factors were also identified to be indirectly involved in regulation of gene expression under stress conditions.

In conclusion, these 125 potential target genes are involved in various biological processes such as metabolism, stress response, ion transport, transcription regulation, and signal transduction. The information of target genes was listed in Supplemental Table S1.

### Validation of new cloned miRNAs by semi-quantitative RT-PCR

**Fig. 3:**
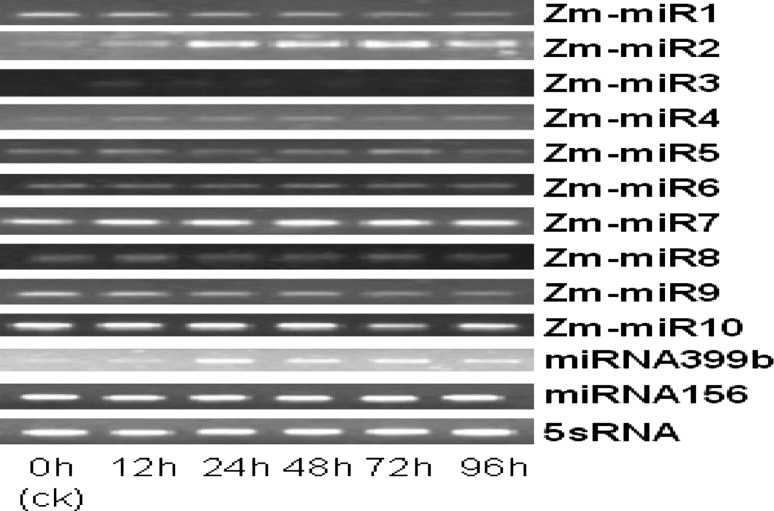
Altered expression of miRNAs after 0–96 h low phosphorus stress shown by semi-quantitative RT-PCR

To confirm the existence of the newly cloned miRNAs and validate their temporal expression trends obtained from maize roots under low phosphorus stress (Fig. 3). Semi-quantitative RT-PCR technology was used. It is shown that all the identified miRNAs were consistent with the previous computational prediction. 7 miRNAs showed several different expression patterns and could be categorized into four types. Zma-miR2 and miRNA399b showed considerably increasing, Zma-miR1 and Zma-miR9 showed gradually and substantially decreasing but Zma-miR3 moderately reduced. However, Zma-miR5 was first repressed and then increased, and miRNA156, Zma-miR7 and Zma-miR10 showed no significant changes and were similar to 5sRNA. The expressional differences of the novel miRNAs were shown in (Fig. 3).

### Analysis of miRNA399b, Zma-miR3 and their target genes by real-time PCR

**Fig. 4:**
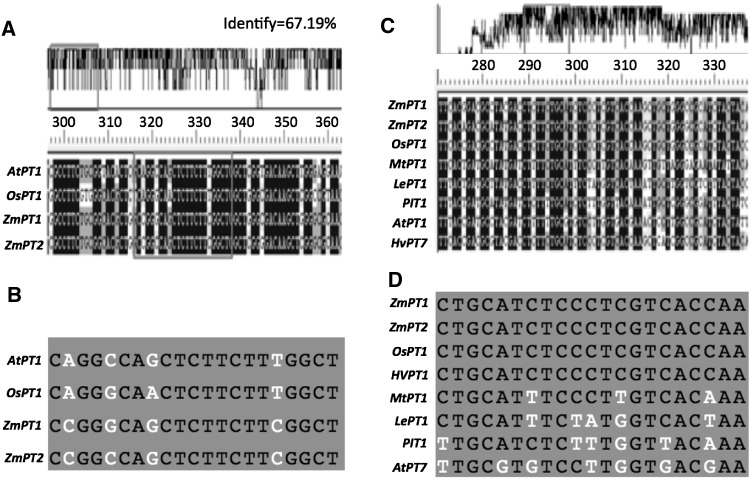
**a** Homology of inorganic phosphate proteins among *Arabidopsis*, rice, and maize. **b** The known target genes’ binding sites in rice and *Arabidopsis* and the putative binding sites for miRNA399b in maize. **c** The homology of inorganic phosphate proteins among some plant species. **d** The putative target genes’ binding sites for Zm-miR3 in gramineous plants

MiRNA399b negative regulated an inorganic Pi transport protein (At3g54700.1, Os08g45000.1) in *Arabidopsis* and rice, respectively, which played an important role in response to low phosphorus stress. Our microarray analysis also demonstrated that two maize inorganic transporters ZmPT1 and ZmPT2 were induced by low phosphorus stress (data not shown) in maize. It seems that miRNA399b might modulate the expression of Pi transport proteins. We used DNAMAN software for BLASTN search for the homology of inorganic Pi transport proteins in *Arabidopsis*, rice and maize, and the possible binding sites for miRNA399b and Zma-miR3 were also analyzed. Our results showed that there is 67.19 % similarity in the three inorganic Pi transport proteins (Fig. 4a). Our results showed that the Zma-miR3 binding site is different from that of miRNA399b in maize. The binding region for miRNA399b is conserved among monocotyledon which is conserved among rice, maize and *Arabidopsis* (Fig. 4b). It is likely that Zma-miR3 binding region is conserved only in gramineous species.

**Fig. 5:**
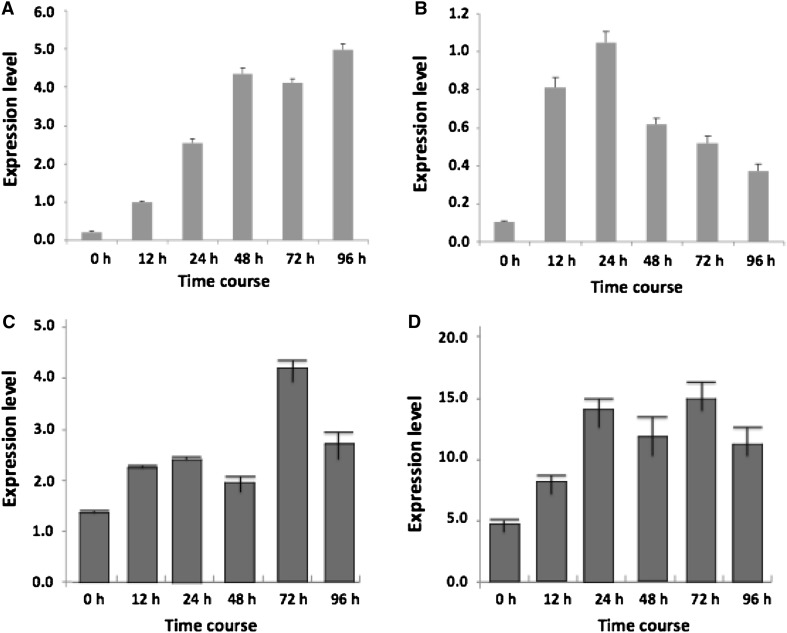
The expression patterns of miRNA399 (**a**), Zm-miRNA3 (**b**), and *ZmPT1* (**c**), and *ZmPT2* (**d**) demonstrated by real-time PCR

Real-time PCR was performed to examine the expression patterns of miRNA399b and Zma-miR3 and their same target genes (*ZmPT1* and *ZmPT2*) were also analyzed. It is revealed that both miRNA399b and Zma-miR3 were induced by low phosphorus stress. But their expression levels were different. MiRNA399b showed a larger extent than Zma-miR3, which was consistent with the results of qRT-PCR analysis. However, their target genes showed the comparable expression levels induced by low phosphorus stress (Fig. 5). It is suggested that the two maize inorganic Pi transporters may have similar functions in response to low phosphorus stress in maize.

## Discussion

MiRNA cloning is extensively used for miRNA detection both in plants and animals as a classic method [17–19]. It can detect the known miRNAs, and more importantly, it can discover the novel, species-specific or stress-regulated specific miRNAs. Thousands of plant miRNAs have been discovered and deposited in miRBase. However, many stress-regulated specific miRNAs were yet detected. The studies of miRNAs mainly concentrated on model plants such as rice and *Arabidopsis* [17–20] and predicted by computational methods. Recently, more efforts have been made to detect maize miRNAs and identify their functions [21, 22]. Our knowledge in the field of stress-regulated maize miRNAs is still limited.

### Known and newly cloned miRNA in maize

In this study, 12 miRNAs were cloned from maize seedling roots under low phosphorus stress, including ten new predicted miRNAs, and their conservation were analyzed by NCBI gramineous genomic database. No miRNAs were conserved with the exception of Zma-miR3. It seems to be inconsistent with the reports that most miRNAs are conserved in plants [11]. However, reports on species-, tissue- and stress-specific miRNAs have shown that some of those miRNAs involved in tissue development and stress response might be unique [23]. In addition, the sequence of Zma-miR9 is almost identical with maize miRNA169 but different from the others and no well matched sequences were found in other species by searching all the miRNA169 family sequences available in miRBase. So it is possible that our newly cloned maize miRNAs are tissue-or stress-specific and Zma-miR9 may be a new member of maize miRNA169 family and maize-specific miRNAs in our cloned miRNA.

### Target prediction of candidate miRNAs and functional analysis

The knowledge of target gene’s function is helpful if we are going to decide whether the further study of candidate miRNAs is necessary. Earlier research in Arabidopsis has demonstrated that target genes of candidate miRNAs were predominantly transcription factors [18] and most miRNAs were involved in many diverse biological processes [24]. In our study, 125 target genes were predicted and the functions of these genes may be classified in several major groups. One major group (transcription factors) accounts for 30.6 % of all target genes, including *bZIP*, *WRKY*, zinc finger, *bHLH*, and *SBP*, which are involved in stress response [25–27], and of which, several members of *bZIP* and *bHLH* families are directly responsible for low phosphorus stress response [28]. Another major group contains metabolism-related proteins such as glycosyl transferase, glyceraldehyde 3 phosphate dehydrogenase (*GAPDH*), sucrose phosphate synthase, serine-threonine/tyrosine-protein kinase, some of which have been participated in stress response pathways [29]. For example, *GAPDH* were involved in stress response in *Arabidopsis* and potato [30, 31] by mediating ROS signaling, a vital pathway under stress conditions in plants [32]. However, no existing studies propose that metabolism-related target genes be directly involved in low phosphorus stress response.

The next group of target genes are those stress modulation proteins such as manganese superoxide dismutase (SOD-3), thioredoxin, glutathione S-transferase, and heat shock protein [33, 34]. SOD-3 is targeted by two of our newly cloned miRNAs, but there is no evidence suggesting that they are transcribed from the same miRNA gene. In *Arabidopsis*, miRNA398 is involved in regulation of Cu–Zn superoxide dismutase 1 and 2 (CSD1 and CSD2) under oxidative stress. MiRNA398 is transcriptionally down-regulated to alleviate its suppression of *CSD1* and *CSD2* genes. Thus, its down-regulation caused the accumulation of *CSD1* and *CSD2* mRNAs, which were important for plant stress resistance [8]. So it is likely that the two newly cloned miRNAs regulated the same manganese SOD gene which might be involved in low phosphorus stress response.

### The expression patterns of maize miRNA399b, Zma-miR3, *ZmPT1*, and *ZmPT2*

In the long course of evolution, plants have developed highly specialized and complicated molecular networks to counter low phosphorus stress; the activation of specific stress response-genes seems to be a universal adaptation strategy. Among those target genes, inorganic Pi transporters are particularly important, because they have been extensively studied in model plants such as rice and *Arabidopsis* [35–37]. Recently, miRNA399 have been demonstrated in *Arabidopsis* and rice which is involved in Pi starvation responses [7, 38–40]. The expression patterns of our cloned maize miRNA399b showed that it is induced by low phosphorus stress and the result is consistent with those obtained in *Arabidopsis* [38]. We speculated that the genes *ZmPT1* and *ZmPT2* are targeted by maize miRNA399b, using the binding region of inorganic Pi transporters conserved in maize, rice, and *Arabidopsis* (Fig. 4c, d). The results of real-time PCR analysis showed that they were induced by low phosphorus stress and were consistent with the previous microarray analysis data (data no shown).

In *Arabidopsis*, another protein, ubiquitin-conjugating E2, has been reported to be involved with Pi regulation process neglect regulated by miRNA399 in *Arabidopsis* and rice [38], under phosphorus starvation, the expression of miRNA399 is induced and E2 expression is suppressed. It would alter the root architecture, which was in charge of maintaining the appropriate Pi level in plants [8, 38].

## Conclusions

The aim of this study was to identify the candidate miRNAs involved in low phosphorus stress response in maize seedling. Twelve miRNAs were discovered, including ten newly cloned and two conserved (miRNA156 and miRNA399). Among the ten novel miRNAs, except for Zm-miR2 is conserved in maize, rice, wheat, and sorghum, the others might be specific to maize. Zm-miR3 is similar to the known miRNA399, regulating the inorganic Pi transporters. In addition, Zma-miR9 may be a new member of maize miRNA169 family and maize specific miRNAs in our miRNA library. Target prediction of candidate miRNAs and functional analysis showed that some of them are directly or indirectly involved in stress response. The expression patterns of miRNAs and their target genes were analyzed by stem RT-PCR and real-time PCR. It provided an indication that miRNA399 was involved in maize seedling roots response to low phosphorus stress and showed an *Arabidopsis* or rice like regulation mechanism. Other species or tissue specific miRNAs might also be directly or indirectly involved in this process.

## Electronic supplementary material

Below is the link to the electronic supplementary material.

Supplementary material 1 (DOC 32 kb)

Supplementary material 2 (DOC 63 kb)

Supplementary material 3 (XLS 43 kb)
