# Supplementary material for: Cloning and characterization of miRNAs from maize seedling roots under low phosphorus stress
Source: Mol Biol Rep. 2012 May 5;39(8):8137–46. doi: 10.1007/s11033-012-1661-5 (PMC3383953; doi:10.1007/s11033-012-1661-5)
Supplement: Supplementary file 1 — Supplementary material 1 (DOC 32 kb) [file 11033_2012_1661_MOESM1_ESM.doc]

**Supplemental Figure S1**. Predict secondary structures of precursors of novel cloned miRNA sequences.

Pri-Zm-miRNA1 (MIR7): TTGTTTGGAATTATAATCTGC G = -83.20 kcal/mol

U C A G AG

AGGGC UGUUUGUUUGGAAUUAUAAUCUGCCUA AUUAUAUAAUCUAA UUAUUUUGAGCUA \

UCCCG ACAAACAAACCUUAAUAUUAGACGGGU UAAUAUAUUAGAUU AAUAAAACUUGAU U

U^ A C G CG

C C A GC

AGGGCUUGUUUGUUUGGAAUUAUAAUCUGCC AGAUUAUAUAAUCUAA UUAUUUUGA CUA \

UCCCGGACAAACAAACCUUAAUAUUAGACGG UUUAAUAUAUUAGAUU AAUAAAACU GAU A

A C C UC

A A

UGUUUGUUUGG AUUAUAAUCUGCUUAUAUUAUAUAAUCUAACUU A

ACAAACAAACU UAAUAUUAGACGAGUAUAAUAUAUUAGAUUGAA A

C U

C A G AG

AGGGC UGUUUGUUUGGAAUUAUAAUCUGCCUA AUUAUAUAAUCUAA UUAUUUUGAGCUA \

UCCCG ACAAACAAACCUUAAUAUUAGACGGGU UAAUAUAUUAGAUU AAUAAAACUUGAU U

A C G CG

U A C ---- C C

GUUUGU UGG AUUAUAAUCUGCUCAGAUUAUAUAAUU AACUUAUUU UGAA UAA \

CAAACG ACC UAAUAUUAGACGGGUUUAAUAUAUUAG UUGAAUAAA AUUU AUU A

C C A UAAA U C

Pri-Zm-miRNA2 (MIR20):AGAGGGGGATTGGAGGGGATT G = -67.40 kcal/mol

- A - A C U A

ACACCAAU UAGAGGGGG AUUGGAGGGG UUAAAU CCC UCUAGUCA A

UGUGGUUA GUCUCCCCC UAACCUCUCC AAUUUA GGG GGAUCAGU A

A G U A A C A

Pri-Zm-miRNA3 (MIR41):TTGGTGACCAGGGAAATGGAG G = -64.70 kcal/mol

U A AG CCCC

UUGGUGACCAGGGAAAUGGAGGGG UCCAUGGGG GAAU C

AACCACUGGUCCCUUUGCCUCCCC AGGUACCCC CUUA U

A C A- UCGU

G CCUUGC

CUGGUUUGGUGACCAGGGAAAUGGAGG GAUCCAUGGG \

GAUUAAACCACUGGUCUCUUUACCUCC CUAGGUACCC U

A CACUUA

G A G CUCC

UCUU AGGGCUG UUUGGUGACCAGGGAAAUGGAGGGG AUUC \

GGAA UCCCGAU AAACUACUGGUCCUUUUACCUCCCC UAAG C

G C - UACC

Pri-Zm-miRNA4 (MIR64):TGAGAGAATGGTATAATCACA G = -61.40 kcal/mol

G -- A --- G G AUU U .-AACUUU| UUAUCUAUCU A

AGG UAAUU UGAUUAUACC CUCU AC UUGCA GUGAUUUUACCC CGUUUUUA GUAAUU GUUUUA A

UCC AUUAA ACUAAUAUGG GAGA UG AAUGU UACUAAAAUGGG GUAAAAAU CGUUAG CGAAGU A

G UA C UAA G G --- U \ ------^ UCGC------ A

Pri-Zm-miRNA5 (MIR70):TGAAGGGGATTGGAGAGGAT G =-42.10 kcal/mol

UGC GG G U-- .-AA| A CAA

GAAG AUUGAAG GGAU GGAGAGGAUUA UCCCUUC UAUU U

UUUC UAAUUUU CCUA CCUCUCUUAGU AGGGAAG AUAA A

AAA A- A UCU \ --^ G AUU

Pri-Zm-miRNA6 (MIR81):TCTAAAATGAGTGGTGCTGAT G = -69.80 kcal/mol

C U -| C AUCAU A ACAAAUA ACCACUACA

ACAUAUUAAAUCA CACUA UCAU UUAGAUCUAAUGUCUCUAGUUG UAUA UUU ACAUCUUCC C

UGUAUAAUUUAGU GUGGU AGUA AAUCUAGAUUACAGAGAUCAAC AUAU AAA UGUGGGAGG C

A C G^ A AAACU A CGAUUCC CUACACCAC

Pri-Zm-miRNA7 (MIR107):AATATTAGACAGAAAAGTTAG G = -24.60 kcal/mol

U| GG AG - AA

CACACUUUGCUUAA UU UUGUUUGA AUUA G

GUGUGAAACGGAUU AA GACAGAUU UAAU A

-^ GA A- A CA

Pri-Zm-miRNA8 (MIR112):GCTCGGCAAAGACTGACGGCC G = -35.00 kcal/mol

A| ACACU AAG C C CAA

GAAG CGGCC AGUC UU GCCGAGUGUC U

UUUC GCCGG UCAG AA CGGCUCGCGG C

A^ GACU- CAG - A CUC

Pri-Zm-miRNA9:TAGCCAGGGATGATTTGCCTG G = -62.00 kcal/mol

A- - CC - C AGA .-GAA| AC

GAGUAAGAG CA CU GGUAG CAGGGAUGAUUUGCC GU UC CAGU A

UUCAUUCUC GU GA CCAUC GUCCCUACUAAACGG CA AG GUUA G

CG A AA G A CCA \ ---^ CG

Pri-Zm-miRNA10: TTCTGATGTTCATGAGCTGGCT G = -41.30 kcal/mol

CAC| G G G G UC G UG AAGAUUG U AUCGCAA

GU GUC UGGUC AG UCGUGG GCG CA GGAGA GC CAUGGG \

CA CGG AUCGG UC AGUACU UGU GU UCUCU CG GUACCU C

CGA^ G - - G -- A CU AUAAGGA U CUUCUUC
