# Supplementary material for: Cloning and characterization of miRNAs from maize seedling roots under low phosphorus stress
Source: Mol Biol Rep. 2012 May 5;39(8):8137–46. doi: 10.1007/s11033-012-1661-5 (PMC3383953; doi:10.1007/s11033-012-1661-5)

**Supplemental Figure S2.** Fold-back structures of conserved sequences of Zm-miR2 in maize, rice, wheat and Sorghum


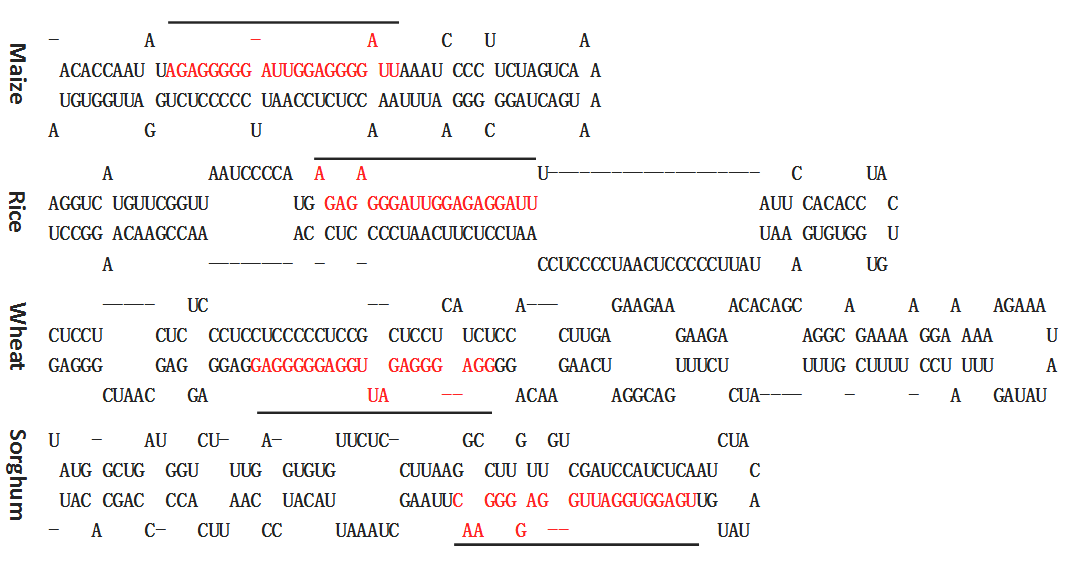

Supplement: Supplementary file 2 — Supplementary material 2 (DOC 63 kb) [file 11033_2012_1661_MOESM2_ESM.doc]
